# Autophagic flux modulates tumor heterogeneity and lineage plasticity in SCLC

**DOI:** 10.3389/fonc.2024.1509183

**Published:** 2025-01-09

**Authors:** Yujie Hao, Mingchen Li, Wenxu Liu, Zhenyi Ma, Zhe Liu

**Affiliations:** ^1^ Department of Immunology, School of Basic Medical Sciences, Tianjin Medical University, Tianjin, China; ^2^ Zhejiang Key Laboratory of Medical Epigenetics, Department of Cell Biology, School of Basic Medical Sciences, Hangzhou Normal University, Hangzhou, China; ^3^ Collaborative Innovation Center for Cancer Personalized Medicine, Nanjing Medical University, Nanjing, China

**Keywords:** autophagic flux, small cell lung cancer, SCLC, lineage transition, heterogeneity, plasticity, genetically engineered mouse model

## Abstract

**Introduction:**

Small cell lung cancer (SCLC) is characterized by significant heterogeneity and plasticity, contributing to its aggressive progression and therapy resistance. Autophagy, a conserved cellular process, is implicated in many cancers, but its role in SCLC remains unclear.

**Methods:**

Using a genetically engineered mouse model (*Rb1^fl/fl^*; *Trp53^fl/fl^*; GFP-LC3-RFP-LC3△G), we tracked autophagic flux *in vivo* to investigate its effects on SCLC biology. Additional *in vitro* experiments were conducted to modulate autophagic flux in NE and non-NE SCLC cell lines.

**Results:**

Tumor subpopulations with high autophagic flux displayed increased proliferation, enhanced metastatic potential, and neuroendocrine (NE) characteristics. Conversely, low-autophagic flux subpopulations exhibited immune-related signals and non-NE traits. *In vitro*, increasing autophagy induced NE features in non-NE cell lines, while autophagy inhibition in NE cell lines promoted non-NE characteristics.

**Discussion:**

This study provides a novel model for investigating autophagy *in vivo* and underscores its critical role in driving SCLC heterogeneity and plasticity, offering potential therapeutic insights.

## 1 Introduction

Lung cancers are generally classified into two categories: small cell lung cancer (SCLC) and non-small cell lung cancer (NSCLC). Small cell lung cancer accounts for approximately 13%–15% of all lung cancer cases and is one of the deadliest cancer types (1, 2) due to its rapid growth, early spread, and resistance to treatment (3, 4). Inactivation of the RB1 and TP53 tumor suppressor genes is observed in nearly all human SCLC cases (3, 5, 6), which led to the development of the *Rb1^fl/fl^; Trp53^fl/fl^* (*RP*) genetically engineered mouse model (GEMM) for SCLC. In this model, Rb1 and Trp53 can be inactivated in lung epithelial cells using an adenovirus that expresses Cre recombinase (7).

Small cell lung cancer, was once thought to be a histologically uniform disease but is now recognized for its profound heterogeneity and plasticity, both of which drive its notorious resistance to therapy (8–10). Based on the expression of four transcription factors, SCLC is classified into the ASCL1 (SCLC-A), NEUROD1 (SCLC-N), YAP1 (SCLC-Y), and POU2F3 (SCLC-P) subtypes (11). Additionally, an inflamed subtype (SCLC-I) has been identified, which has shown enhanced responses to immunotherapy (12). While SCLC-A and SCLC-N are neuroendocrine (NE) subtypes, the others are categorized as non-neuroendocrine (non-NE) subtypes. Importantly, recent evidence challenges the notion that these subtypes are static; SCLC displays considerable plasticity, with tumor cells able to transition between NE and non-NE states in response to environmental cues or therapeutic pressure (13). This adaptability is exemplified by the coexistence of small NE cells and larger, mesenchymal-like non-NE cells, as demonstrated in both patient samples and mouse models (14). A deeper understanding of SCLC’s heterogeneity and plasticity is essential for addressing its drug resistance and for developing more effective therapeutic strategies.

Autophagy, a process that recycles and degrades cellular components, is essential for maintaining cell health and plays multiple roles in various biological functions (15–17). While much research has been conducted on autophagy in NSCLC, its role in SCLC remains understudied (18–20). To assess autophagic flux, a fluorescent probe (GFP-LC3-RFP-LC3△G) was developed (21). The GFP-LC3 component is degraded via autophagy, while RFP-LC3△G remains an internal control, allowing the quantification of autophagic flux based on the GFP/RFP signal ratio.

In this study, we established transgenic mice expressing this fluorescent probe and crossed them with the *RP* mouse model to detect autophagic flux *in vivo*. We examined how autophagy affects SCLC growth, metastasis, and cell differentiation, and revealed that autophagy significantly influences SCLC heterogeneity and plasticity.

## 2 Materials and methods

### 2.1 Generation of *Rb1^fl/fl^; Trp53^fl/fl^;* GFP-LC3 -RFP-LC3△G mice

GFP-LC3-RFP-LC3ΔG-knockin mice were generated in collaboration with ViewSolid Biotech (Beijing, China), via the PiggyBac transposon system for efficient gene transfer. This system enables the “cut-and-paste” mechanism, where the transposase enzyme recognizes inverted terminal repeats (ITR) at both ends of the transposon vector, excises the sequence, and inserts it into a TTAA site on the chromosome. The gene element was inserted between the ITR sequences of the PiggyBac system, and co-injected into mouse zygotes to create transgenic mice. The pCAG-Map1lc3b-intron-BGHpa plasmid was linearized via PvuI before microinjection, and PCR was used to identify successful integration in the mice three weeks after birth.

*Rb1^fl/fl^; Trp53^fl/fl^* (*RP*) mice were kindly provided by Dr. Hongbin Ji. The GFP-LC3-RFP-LC3ΔG-knockin mice were then crossed with *RP* mice to produce *Rb1^fl/fl^;Trp53^fl/fl^;*GFP-LC3-RFP-LC3△G (*RP△G*) mice. At 6–8 weeks of age, *RP△G* mice were anesthetized and administered Adenovirus CMV-Cre recombinase (Ad-Cre, 2.5×10^7^ PFU) via intratracheal intubation, allowing Cre-lox mediated recombination of floxed alleles. Tumor tissues were collected 6–8 months after adenoviral infection. The genotyping primers used are listed in **Supplementary Tables 1**-**3**, and all the reagents used are detailed in **Supplementary Table 4**.

All animal experiments were approved by the Animal Care and Use Committee of Tianjin Medical University (TMUaMEC2021058) and complied with national guidelines for the ethical treatment of experimental animals.

### 2.2 Immunofluorescence

Tumor tissues were fixed in 4% paraformaldehyde at 4°C overnight, washed in cold PBS and dehydrated in 25% and 35% sucrose solutions. The tissues were then embedded in optimal cutting temperature compound and sectioned into 8 μm slices for staining. The frozen sections were thawed and washed three times with 1× PBS -T for 5 minutes each. After washing, 50 µL of DAPI staining solution was added, and the sections were covered with clean coverslips. During the GFP/RFP fluorescence analysis, consistent exposure time and intensity were maintained. Fluorescence was analyzed using a laser scanning confocal microscope.

### 2.3 Primary tumor cell isolation

Lung tumor nodules from the mice were mechanically separated, followed by enzymatic digestion using a tumor dissociation kit at 37°C for 45 minutes to obtain a single-cell suspension. Each lung was treated with 5 mL of digestion media, and the process was halted by adding 5 mL of HBSS+ per milliliter of enzyme solution. The HBSS+ solution was prepared by adding 5 mL of 1 M HEPES, 10 mL of fetal bovine serum (FBS), and 5 mL of penicillin-streptomycin-glutamine (100X) to 500 mL of HBSS medium. After digestion, the cells were centrifuged at 600 rcf for 5 minutes at 4°C and treated with 3 mL of 100 U/mL DNaseI for 15 minutes. The cells were then rinsed with HBSS+ buffer through a 100 μm filter, centrifuged, and treated with 2 mL red blood cell lysis buffer to remove remaining blood cells. Finally, the cells were washed with cold HBSS+ and resuspended in PBS supplemented with 2% FBS for further analysis.

### 2.4 Flow cytometry

Dissociated lung tumor cells were washed with PBS + 2% FBS and stained with anti-EPCAM and anti-DAPI antibodies. Cell sorting was conducted using a BD FACS Aria II cytometer, and the data were analyzed using FlowJo software.

### 2.5 Western blot

Proteins were extracted and separated by SDS-PAGE before being transferred onto a PVDF membrane. The membranes were blocked with 5% non-fat milk for 1 hour at room temperature, followed by overnight incubation at 4°C with primary antibodies. After being washed with TBS-T (three times for 5 minutes each), the membranes were incubated with secondary antibodies at room temperature for 1 hour, followed by additional washes. Protein signals were detected via enhanced chemiluminescence. The details of the antibodies and reagents used are listed in **Supplementary Table 4**.

Band intensities were quantified using ImageJ software, where the signal intensity was proportional to the target protein concentration. Protein levels were normalized to GAPDH to account for differences in sample loading. The LC3B-II/LC3B-I ratio was calculated as an indicator of autophagic flux. Statistical analysis was performed using GraphPad Prism, with differences assessed by one-way ANOVA followed by multiple comparisons (p < 0.05).

### 2.6 Bulk RNA-seq data analysis

Total RNA was extracted from tumor tissues or cell lines using TRIzol reagent, following the manufacturer’s protocol. Sequencing was performed by Lianchuan Biology, and bioinformatic analysis was conducted using the OmicStudio platform. Differential expression analysis was performed using DESeq2, and genes with an adjusted P- value ≤ 0.05 and a |log2-fold change| ≥ 1 were considered differentially expressed. Volcano plots of differentially expressed genes were generated using the ggplot2 and ggrepel R packages. GO term enrichment and KEGG pathway analyses were performed using clusterProfiler. GSEA was conducted with Seurat, clusterProfiler, and enrichplot. The sequencing data presented in this study are listed in **Supplementary Table 4**.

### 2.7 Correlation analysis

**Figure 1:**
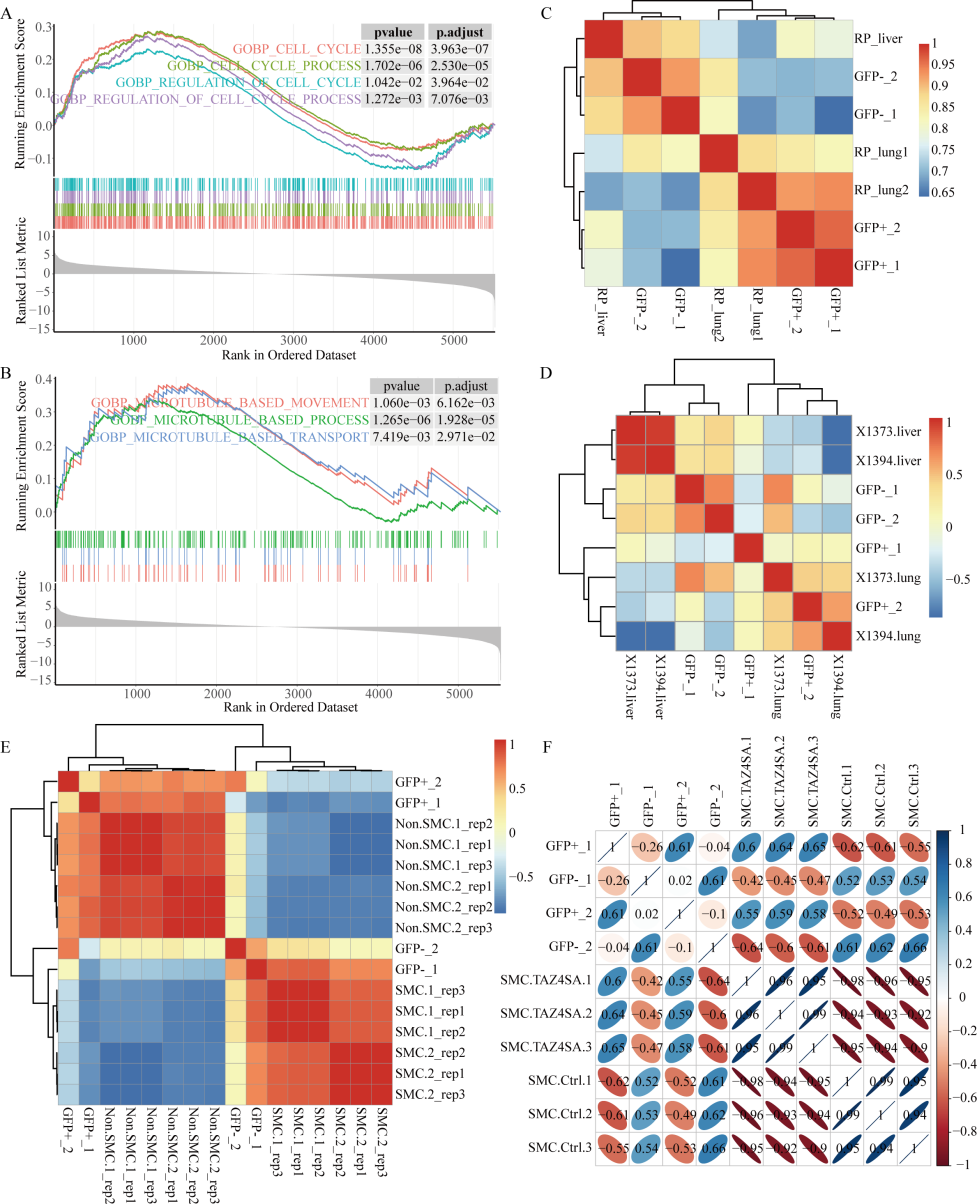
The GFP-negative subpopulation exhibits enhanced proliferation and metastatic potential. **(A)** GSEA revealed significant enrichment of cell cycle-related signaling pathways in the GFP-negative subpopulation. **(B)** GSEA revealed enrichment of microtubule-related pathways in the GFP-negative subpopulation. **(C–F)** Spearman correlation analysis was conducted to assess the associations between sequencing data from four samples, representing two subpopulations, and other sequencing data from *RP* GEMMs. **(C)** Heatmap displaying correlation clustering analysis based on sequencing data from primary lung tumors and metastatic liver tumors in *RP* mice. **(D)** Heatmap displaying correlation clustering analysis using data from primary lung tumors and metastatic liver tumors from two *RPR2* mouse models, X1373 and X1394, sourced from GSE228333. **(E)** Heatmap showing correlation clustering analysis of SCLC-metastasizing cells (SMCs) and non-SCLC-metastasizing cells (non-SMCs) using *RP* mouse model data from GSE158293. **(F)** Correlation clustering analysis of SMC and ectopic TAZ expression in SMC (SMC.TAZ4ASA), which has been shown to mitigate SCLC metastasis, using data from GSE158290.

**Figure 2:**
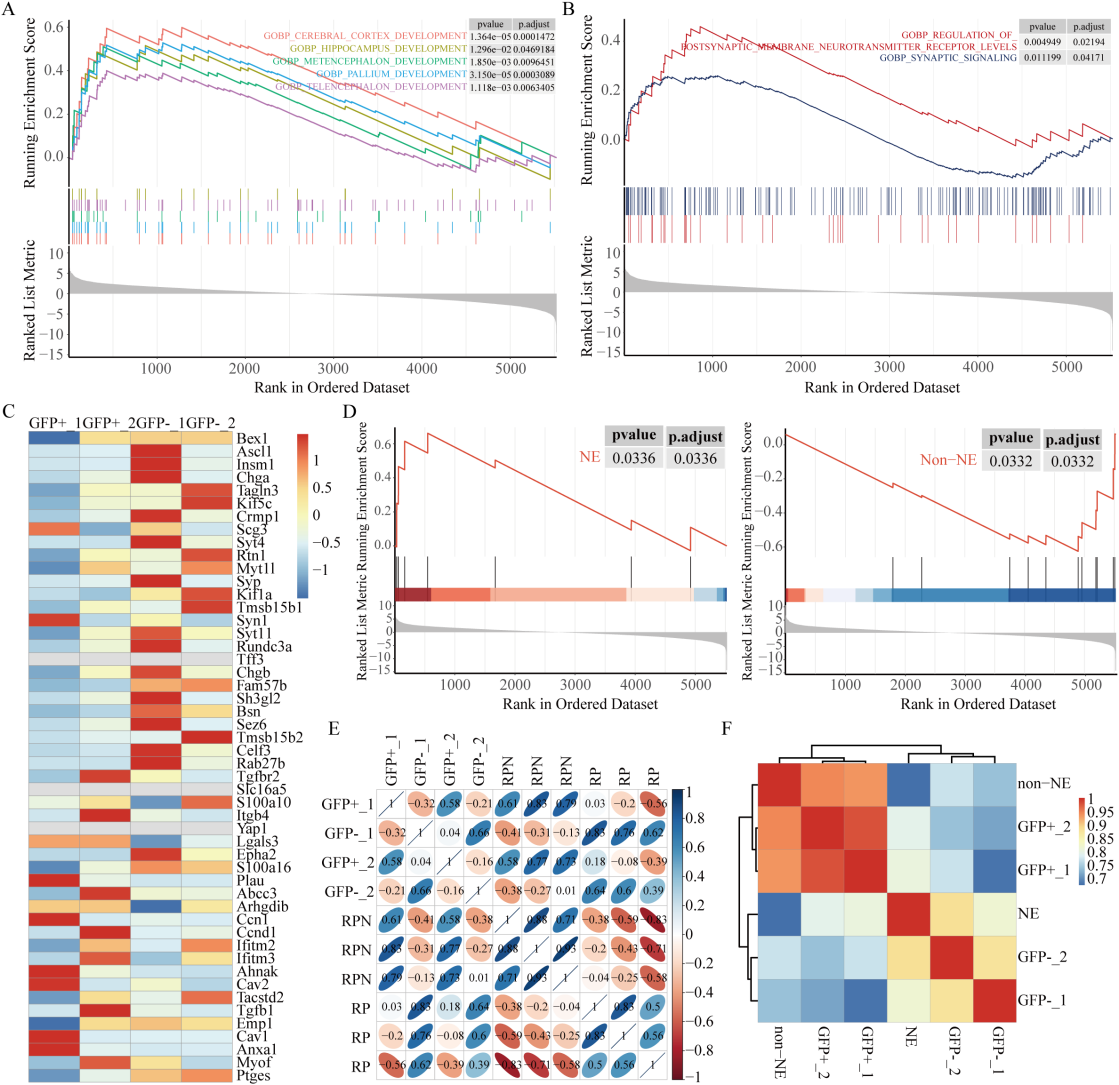
High autophagic flux is associated with neuroendocrine signature enrichment. **(A)** GSEA revealed significant enrichment of brain development-related pathways in the GFP-negative subpopulation. **(B)** GSEA revealed high expression of synaptic pathways associated with the NE phenotype in the GFP-negative subpopulation. **(C)** Heatmap of 50 NE and non-NE genes (38). **(D)** GSEA comparing the GFP-negative subpopulation with the GFP-positive subpopulation. **(E)** Correlation clustering analysis of *RP* and *RPN* (*RP*; *Nkx2-1^fl/fl^*, reduced NE-related genes) mice, using data from GSE183371. **(F)** Correlation analysis using time-series scRNA-seq data from RPM-Cas9 mice (GSE149179), clustered based on NE and non-NE features. The clustered data were then correlated with our own dataset.

For correlation analysis, we used data from the following datasets: GSE228333 (**Figure 1D**), GSE158293 (**Figure 1E**), GSE158290 (**Figure 1F**), GSE183371 (**Figure 2E**), and GSE149179 (**Figure 2F**). To correct for batch effects, the combat function from the sva R package was applied. Correlation coefficients were calculated using Spearman’s rank correlation method, with analysis supported by several R packages, including corrplot, ggplot2, ggcorrplot, vcd, psych, and ggrepel.

### 2.8 Trehalose and bafilomycin A1 treatment

H841 (non-NE, SCLC-Y) and H1048 (non-NE, SCLC-P) cells were cultured in 6 cm dishes until they reached approximately 70% confluence before the experiments commenced. The experimental groups were treated with trehalose (Tre) at concentrations of 50 mM, 100 mM, and 200 mM in the culture medium, whereas the control group received normal culture medium. After 12 hours of treatment, the cells were harvested for subsequent experiments.

H209 (NE, SCLC-A) and H1092 (NE, SCLC-A) cells were similarly cultured to approximately 70% confluence in 6 cm dishes. Bafilomycin A1 (BafA1) was dissolved in DMSO at a stock concentration of 1 mM. The experimental groups were treated with BafA1 at various concentrations (250 nM, 500 nM, 1 μM, 2 μM, and 2.5 μM), whereas the control group received culture medium supplemented with 5 μL of DMSO. The cells were collected for further analysis after 24 hours of treatment.

H841 and H1048 cells were cultured in DMEM/F12 supplemented with 10% FBS and 1% insulin-transferrin-selenium (ITS), while DMS114 cells were grown in DMEM supplemented with 10% FBS. H209 cells were maintained in RPMI 1640 medium supplemented with 10% FBS.

## 3 Results

### 3.1 Establishment of a triple-transgenic mouse model for monitoring autophagy in SCLC

To investigate the role of autophagy in SCLC, a GFP-LC3-RFP-LC3ΔG-knockin mouse model was developed to facilitate *in vivo* monitoring of autophagic flux. This model employs a fluorescence probe that is cleaved by endogenous ATG4 proteases, resulting in the formation of GFP-LC3 and RFP-LC3ΔG (21). GFP-LC3 undergoes lipidation, becomes incorporated into autophagosomes, and is subsequently degraded following lysosomal fusion. In contrast, RFP-LC3ΔG, which is unable to undergo lipidation, remains in the cytoplasm as a stable internal control. The inverse ratio of GFP to RFP fluorescence provides a quantitative measure of autophagic flux, allowing for the precise assessment of autophagy dynamics in the context of SCLC.

**Figure 3:**
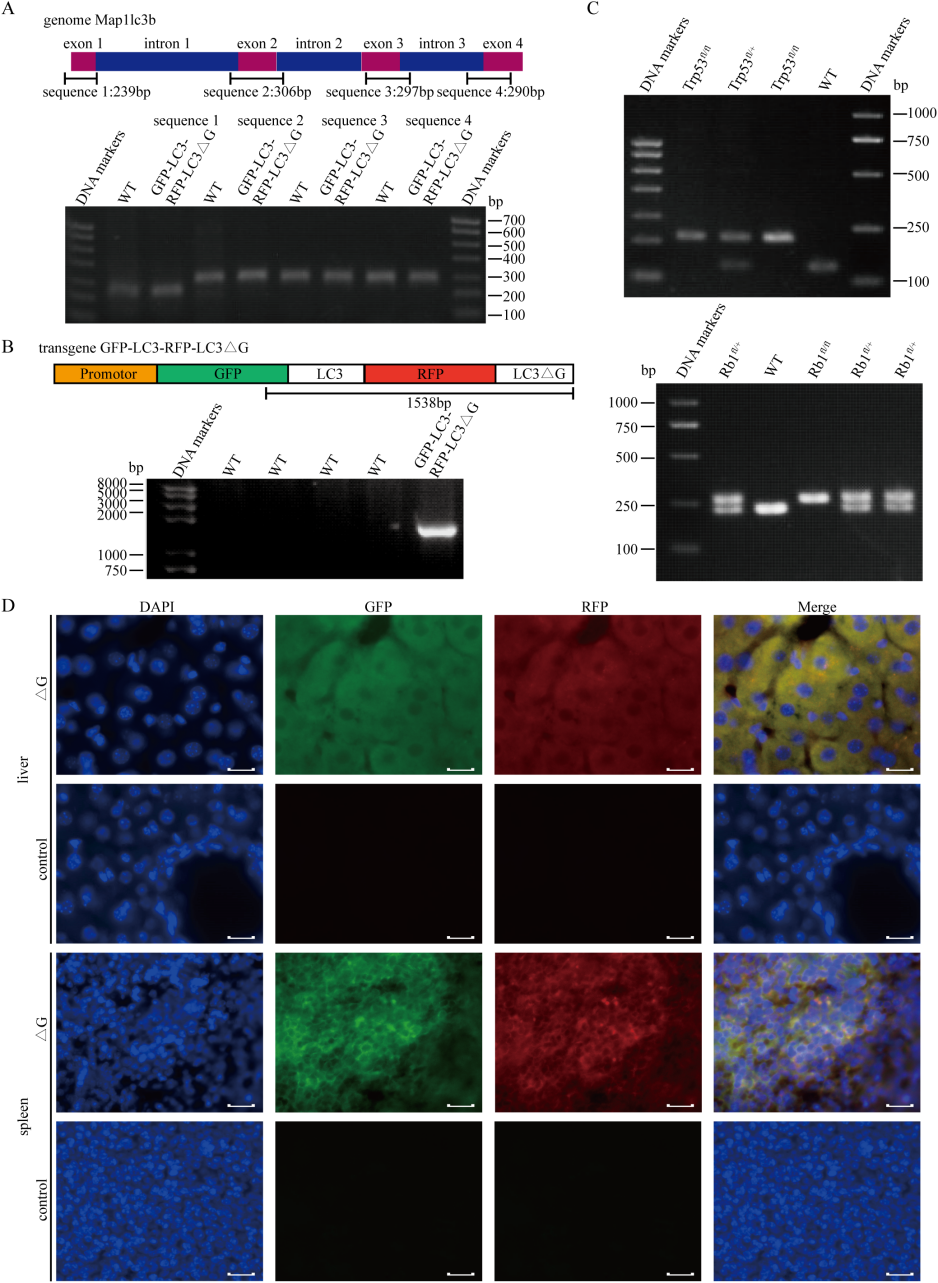
Establishment of the *Rb1^fl/fl^;Trp53^fl/fl^;*GFP-LC3-RFP-LC3△G triple-transgenic mouse model for SCLC. **(A)** PCR-based genotyping of four exonic regions of the endogenous LC3 gene using genomic DNA extracted from mouse tail tissue. **(B)** PCR results confirming the integration of the GFP and RFP sequences into the transgenic mice. **(C)** Upper panel: Genotyping results of *Trp53^fl/fl^* (212 bp), *Trp53^fl/+^* (212 bp/130 bp) and *Trp53^+/+^* (130 bp) mice. Lower panel: Genotyping results of *Rb1^fl/fl^* (283 bp), *Rb1^fl/+^* (283 bp/235 bp) and *Rb1^+/+^* (235 bp) mice. **(D)** Fluorescence imaging of cryosections from liver and spleen tissues of *RPΔG* and *WT* mice. DAPI (blue) indicates nuclear staining, GFP (green) indicates GFP expression, and RFP (red) represents RFP expression. Scale bars: 50 µm.

GFP-LC3-RFP-LC3ΔG-knockin mice were crossed with *Rb1^fl/fl^; Trp53^fl/fl^* (*RP*) mice to establish an animal model exhibiting molecular and histopathological features similar to those observed in human SCLC (7). PCR identification was performed on the mice three weeks after birth. Identification of GFP-LC3-RFP-LC3ΔG-knockin mice was performed in two steps. First, endogenous LC3 was genotyped using four pairs of primers targeting its exon regions, confirming that the ΔG insertion did not disrupt LC3 (**Figure 3A**). Next, a primer pair spanning both GFP and RFP was used to confirm the insertion of the Map1lc3b sequence into the genome (**Figure 3B**). Additionally, genotyping confirmed that the *Trp53^fl/fl^* and *Rb1^fl/fl^* alleles were homozygous (**Figure 3C**).

Fluorescence analysis of tissues from *Rb1^fl/fl^;Trp53^fl/fl^;*GFP-LC3-RFP-LC3△G (*RPΔG*) and wild-type (*WT*) mice revealed distinct GFP and RFP signals in transgenic mice, whereas no such signals were detected in *WT* mice (**Figure 3D**). Small cell lung cancer phenotype tumors formed within eight months following intratracheal injection of an adenovirus carrying Cre recombinase, driven by the CMV promoter, into *RPΔG* mice.

### 3.2 Autophagic flux indicates tumor heterogeneity *in vivo*

**Figure 4:**
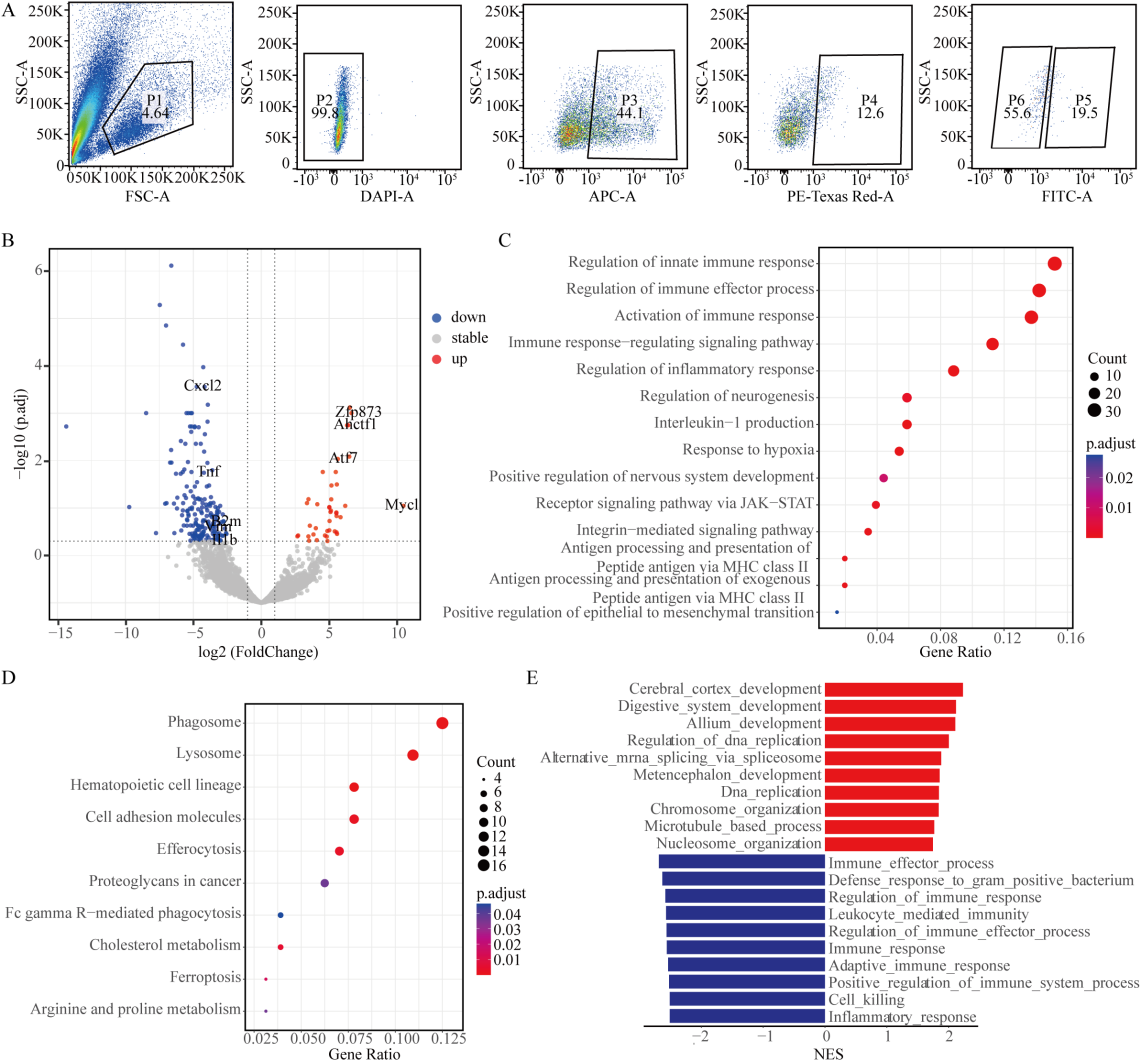
Autophagic flux reveals tumor heterogeneity *in vivo*. **(A)** Flow cytometry analysis of dissociated lung tumor cells from *RP△G* mice. The cells were gated to exclude debris, and the DAPI-negative live cells were isolated. EpCAM-positive cells were further sorted into RFP-positive cells. Based on GFP expression, distinct subpopulations were isolated for subsequent RNA sequencing analysis. **(B)** Volcano plot of differential gene expression analysis for RNA-seq data comparing the GFP-negative subpopulation with the GFP-positive subpopulation. **(C)** Gene Ontology (GO) enrichment analysis of differentially expressed genes. **(D)** KEGG pathway analysis of upregulated genes in the GFP-negative subpopulation. **(E)** Gene set enrichment analysis (GSEA) identifying the top ten pathways upregulated and downregulated in the GFP-negative subpopulation.

The gene expression patterns of the *Rb1^fl/fl^; Trp53^fl/fl^* mouse model closely resemble those of the ASCL1 subtype of human SCLC (11, 22). Previous research confirmed that EpCAM, a cell surface marker, distinguishes ASCL1+ SCLC cells from NEUROD1+ SCLC cells, enabling in-depth molecular characterization of different SCLC subtypes (23). Normal lung tissues from *WT* mice and lung tumor tissues from *RPΔG* mice were harvested, digested into single cells, and cleared of red blood cells. Fluorescence-activated cell sorting (FACS) was utilized to isolate cellular subpopulations. Initially, the cellular debris was removed, followed by isolation of the DAPI-negative live cells. EpCAM-positive and RFP-positive cells were subsequently separated from these live cells, followed by screening for GFP-positive and GFP-negative cells for RNA sequencing (**Figure 4A**). No RFP-positive fluorescence signal was detected in the control group of *WT* mouse cells (**Supplementary Figure S1**).

Spontaneous lung tumors were successfully induced and sequenced in two *RPΔG* mice. Differential transcriptome analysis of the two subpopulations revealed that transcription-related genes, such as *Ahctf1*, *Atf7*, and *Zfp873*, were highly expressed in the GFP-negative (GFP-) subpopulation, which presented increased levels of autophagic flux. *Mycl* showed the most significant difference in expression in the GFP-negative subpopulation (**Figure 4B**). Amplification or high expression of *Mycl* is essential for the development of SCLC-A (3, 10, 24). Elevated levels of immune-related factors, such as *Cxcl2*, *B2m*, *Il1b*, and *Tnf*, were observed in the GFP-positive (GFP+) subpopulation, which exhibited decreased autophagic flux. Additionally, *Vim* was highly expressed in the GFP-positive subpopulation (**Figure 4B**). Gene ontology (GO) analysis revealed that the main pathways that differed between the two subpopulations involved inflammation and immune response-related pathways, as well as neurogenesis, hypoxia-related pathways, and JAK-STAT pathways (**Figure 4C**).

Kyoto Encyclopedia of Genes and Genomes (KEGG) pathway analysis indicated that, the GFP-negative subpopulation presented increased expression of pathways associated with phagosome, lysosome, efferocytosis, and ferroptosis compared with that of the GFP-positive subpopulation (**Figure 4D**). This finding aligns with the characteristic increased autophagic flux in the GFP-negative subpopulation. Furthermore, Gene Set Enrichment Analysis (GSEA) revealed that the top upregulated pathways in the GFP-negative subpopulation involved primarily DNA replication, transcription, and brain development, whereas the GFP-positive subpopulation was enriched in pathways related to inflammation and the immune response (**Figure 4E**). These findings suggest that tumor cells with varying levels of autophagic flux within the same tumor display unique transcriptional characteristics.

### 3.3 Enhanced proliferative and metastatic potential of the GFP-negative subpopulation in SCLC

Small cell lung cancer is characterized by rapid proliferation and a remarkable propensity for metastasis. Approximately 50% of *RP* GEMMs spread to the lymph nodes, liver, spleen, kidneys, and other organs (7, 25). The differences in proliferation and metastatic abilities between the two subpopulations were compared. The GFP-negative subpopulation was enriched with cell cycle-related signaling pathways (**Figure 1A**), along with pathways related to chromatin organization, sister chromatid segregation, DNA replication, and RNA transcription, indicating that the GFP-negative subpopulation has a faster proliferative capacity (**Supplementary Figures S2A–S2C**).

Additionally, the GFP-negative subpopulation was enriched in microtubule-related pathways (**Figure 1B**), prompting further investigation into its metastatic potential. Correlation clustering analysis was performed on the sequencing data from the two subpopulations, and the results were compared with those from *RP* mouse primary lung tumors and metastatic liver tumors. The results indicated that the GFP-negative subpopulation was more similar to the expression pattern of liver metastasis, whereas the GFP-positive subpopulation data were more similar to those of primary lung tumors (**Figure 1C**).

To validate the reliability of these results, sequencing data with metastatic features were selected from the same classic SCLC *RP* GEMMs in the GEO database for comparative analysis. Julie H. Ko et al. (26) provided paired sequencing data of primary lung tumors and liver metastatic tumors in two *Rb1^f/f^;Trp53^f/f^;Rbl2^f/f^* mouse models (**Figure 1D**). Yujuan Jin et al. (27) identified the subpopulations of SCLC metastasizing cells (SMCs) and non-SCLC metastasizing cells (non-SMCs) via the *RP* mouse model, obtaining sequencing data for six pairs of SMC and non-SMC subpopulations (**Figure 1E**). Additionally, ectopic TAZ expression was found to facilitate the reverse transition from SMCs to non-SMCs, alleviating SCLC metastasis (**Figure 1F**). Correlation analysis confirmed that the GFP-negative subpopulation exhibited a greater degree of similarity to tumors with metastatic characteristics.

Furthermore, gene expression analysis of validated regulators involved in the progression and metastasis of SCLC was performed (**Supplementary Figure S2D**). Previous studies have demonstrated that *Max* and *Pten* (28) inhibit SCLC proliferation and are expressed at relatively high levels in the GFP-positive subpopulation. Moreover, the expression levels of *Mki67* and *Pcna* are increased in the GFP-negative subpopulation. The transcription factor NFIB is frequently upregulated in SCLC and plays a pivotal role in the progression, invasion, and metastasis of tumors (29–32). Other studies have highlighted the significant involvement of enhancers of EZH2 (29, 33), DLL3 (34, 35), the axonal markers GAP43 and FEZ1 (36) in the propagation and metastasis of SCLC. Yujuan Jin et al. identified the NCAM^hi^CD44^lo/–^ subpopulation as SMCs (27). The heatmap reveals elevated levels of *Ezh2*, *Dll3*, *Gap43*, *Fez1* and *Ncam1* in the GFP-negative subpopulation, as well as diminished expression of *Cd44*. In conclusion, the GFP-negative subpopulation, which exhibited greater levels of autophagic flux, demonstrated enhanced proliferation and migration abilities compared with those of the GFP-positive subpopulation.

### 3.4 High autophagic flux is associated with neuroendocrine signature enrichment

Recently, neuroendocrine (NE) signatures have been established as significant indicators of SCLC metastasis (37). The GFP-negative subpopulation was found to exhibit greater metastatic potential than the GFP-positive subpopulation, prompting an investigation into potential differences in NE characteristics between the two groups. Pathways related to brain development were highly expressed in the GFP-negative subpopulation as revealed by GSEA (**Figure 2A**). Pathways associated with NE signatures, such as postsynaptic membrane neurotransmitter receptor levels and synaptic signaling, were notably enriched in the GFP-negative subpopulation (**Figure 2B**). The relationship between autophagy and NE signatures in the two subpopulations was assessed by analyzing the expression of 25 NE-related genes and 25 non-NE-related genes in human SCLC cell lines (38). More pronounced NE characteristics were demonstrated by the GFP-negative subpopulation as indicated by the heatmap (**Figure 2C**) and GSEA (**Figure 2D**) analysis of the 50-gene signature.

We further took advantage of Ranran Kong et al. RNA-sequencing data (39) derived from *Rb1^fl/fl^; Trp53^fl/fl^* (*RP*) and *Rb1^fl/fl^; Trp53^fl/fl^; Nkx2-1^fl/fl^* (*RPN*) mouse models. A significant decrease in genes related to nervous system development in *RPN* tumors was revealed through differential transcriptomic analysis of tumors from *RPN* and *RP* mice. Interestingly, the GFP-negative subpopulation is hierarchically closer to the *RP* pattern, whereas the GFP-positive subpopulation clusters with *RPN* (**Figure 2E**). Finally, we exploited single-cell RNA-sequencing data from Ireland et al. (10), obtained from unsorted early-stage tumor cells isolated from RPM-Rosa26-LSL-Cas9-Ires-Gfp (*RPM-Cas9*) mice as they transitioned from days 4 to 21 in culture at six distinct time points. According to the article’s description, cells expressing high levels of Ascl1 and NE markers during days 4–7 were categorized into the NE group, while the remaining cells during days 11–21 were classified as non-NE. Consistent with expectations, the GFP-negative subpopulation aligned hierarchically with the NE pattern (**Figure 2F**). These results suggest that the GFP-negative subpopulation, characterized by high autophagic flux, exhibits more significant NE characteristics, possibly representing the NE subtype in SCLC, whereas the GFP-positive subpopulation, characterized by low autophagic flux, displays non-NE features.

### 3.5 Autophagic flux affects the heterogeneity and plasticity of SCLC *in vitro*

**Figure 5:**
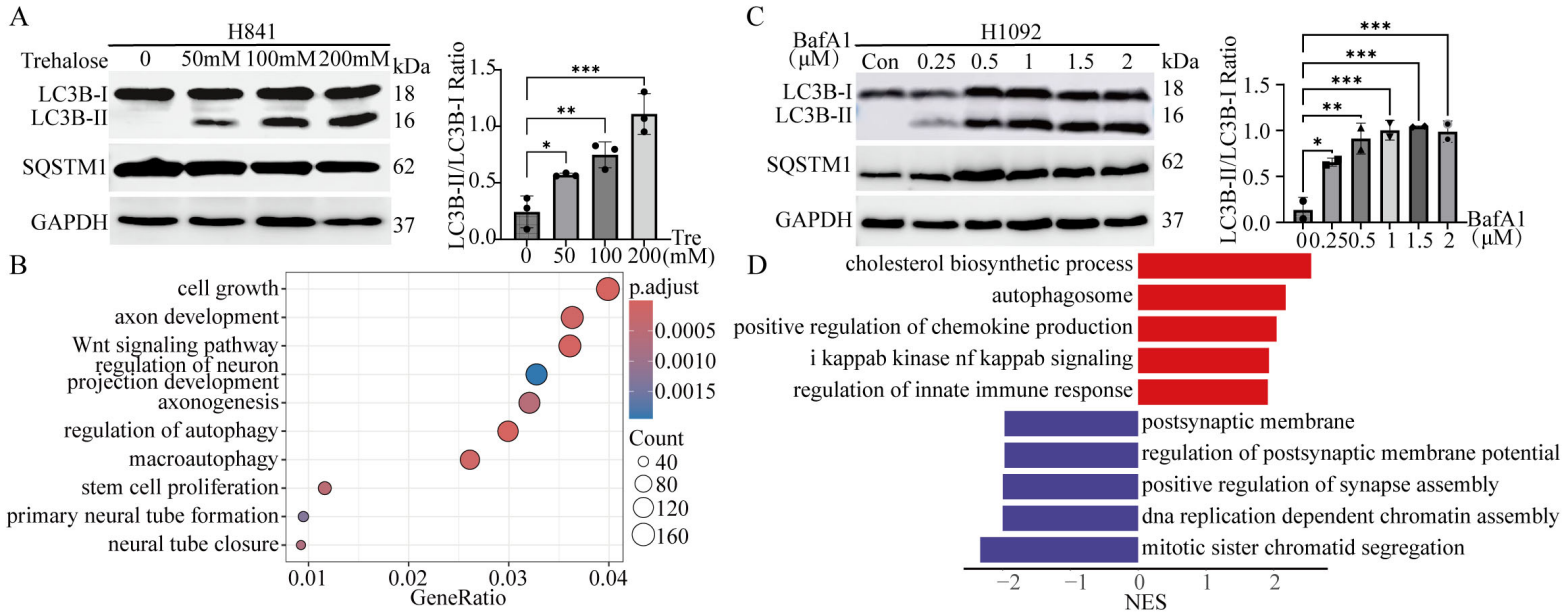
Autophagic flux modulates the heterogeneity of SCLC *in vitro*. **(A)** Left panel: Immunoblot analysis of LC3B and SQSTM1 in H841 cells treated with the autophagy enhancer trehalose (Tre) at the indicated concentrations for 12 hours. GAPDH served as the loading control. Right panel: Quantification of LC3B-II/LC3B-I ratio from the immunoblot shown in the left panel. Results are expressed as mean ± SD from three independent experiments. **(B)** GO analysis revealed signaling pathways enriched in H841 cells treated with trehalose (200 mM for 12 hours). **(C)** Left panel: Immunoblot analysis of LC3B and SQSTM1 in H1092 cells treated with the autophagy inhibitor bafilomycin A1 (BafA1) or DMSO (5 μL) at the indicated concentrations for 24 hours. GAPDH served as the loading control. Right panel: Quantification of LC3B-II/LC3B-I ratio from the immunoblot shown in the left panel. Results are expressed as mean ± SD from two independent experiments. **(D)** GSEA identified signaling pathways enriched in H1092 cells treated with BafA1 (2 μM for 24 hours) compared with control H1092 cells. *: p < 0.05; **: p < 0.01; ***: p < 0.001.

To verify whether autophagy affects the NE characteristics of SCLC, both NE and non-NE SCLC cell lines were treated with autophagy inhibitors or enhancers. In the two non-NE cell lines, H841 and H1048, treatment with the autophagy enhancer trehalose (Tre) (40, 41) at different concentrations for 12 hours resulted in a dose-dependent increase in LC3B-II levels, with a notable elevation in the LC3B-II/LC3B-I ratio (**Figure 5A**). While there was a slight decrease in SQSTM1 levels, no statistically significant change was observed (**Figure 5A**, **Supplementary Figures S3A, S3B**). Sequencing analysis was performed on both control and trehalose-treated H841 cells, and differentially expressed genes were analyzed. Gene ontology (GO) analysis revealed that trehalose-induced autophagy in H841 cells was associated with enrichment in pathways related to cell growth and autophagy, with a notable emphasis on neuroendocrine-related pathways, including the axon development pathway (**Figure 5B**).

Two NE cell lines, H1092 and H209, were treated with various concentrations of the autophagy inhibitor bafilomycin A1 (BafA1) for 24 hours. As previously mentioned, cells treated with BafA1 presented higher levels of LC3B-II than control cells treated with DMSO, with a marked elevation in the LC3B-II/LC3B-I ratio (**Figure 5C**). However, there was only a modest increase in SQSTM1 levels, which did not reach statistical significance (**Figure 5C**, **Supplementary Figures S3C, S3D**). After treatment with 2 μM BafA1, H1092 cells and control cells were sequenced. The results revealed that the inhibition of autophagic flux led to the upregulation of the autophagosome, immune response, and NF-kappaB signaling pathways in H1092 cells, whereas DNA replication and synapse-related pathways were downregulated (**Figure 5D**). These results indicate a decrease in NE characteristics and an increase in non-NE characteristics. Taken together, these findings suggest that the induction of autophagic flux in non-NE cell lines enhances NE characteristics, whereas the suppression of autophagic flux in NE cells reduces NE features and increases non-NE characteristics.

## 4 Discussion

In this study, we developed a novel transgenic mouse model for SCLC, *RPΔG*, which facilitates *in vivo* tracking of autophagic flux. This model enabled the investigation of the role of autophagy in regulating tumor heterogeneity, lineage plasticity, and metastasis in SCLC. Our findings indicate that high autophagic flux is associated with increased tumor proliferation, enhanced metastatic potential, and increased neuroendocrine (NE) characteristics. Conversely, tumor subpopulations with reduced autophagic flux displayed immune-related gene signatures and non-neuroendocrine (non-NE) traits. These observations highlight autophagy as a pivotal regulator of SCLC biology, with the potential to serve as a therapeutic target to control tumor behavior and plasticity.

Small cell lung cancer is recognized as a heterogeneous disease, characterized by the coexistence of NE and non-NE subtypes within the same tumor (11). This heterogeneity, coupled with the ability of tumor cells to transition between the NE and non-NE states, contributes to SCLC aggressiveness and resistance to treatment (9, 10). Research has shown that SCLC tumors can display both small NE cells and larger non-NE cells with mesenchymal-like characteristics (14). Our results support these findings, demonstrating that autophagy modulates phenotypic plasticity. Specifically, increasing autophagy in non-NE cells induced NE characteristics, whereas inhibiting autophagy in NE cells promoted non-NE traits. These findings suggest that autophagic flux acts as a key regulator of the NE/non-NE transition, which is central to SCLC heterogeneity and therapeutic resistance.

Tumor heterogeneity is not only a key feature of SCLC but also closely tied to its metastatic potential. Our data revealed that the tumor subpopulation with high autophagic flux exhibited a greater propensity for metastasis, aligning with a neuroendocrine phenotype (26). These findings are consistent with previous reports that identified a subpopulation of metastasizing SCLC cells (NCAM^hi^CD44^lo/–^) with strong NE characteristics (27). Our study further demonstrated that autophagy can influence the metastatic capabilities of these subpopulations, reinforcing the idea that autophagy drives both SCLC heterogeneity and metastatic behavior.

The role of autophagy in SCLC chemoresistance is equally complex. SCLC tumors often shift from the NE phenotype to the non-NE phenotype during relapse, a state that is associated with increased resistance to chemotherapy (42). This plasticity is driven in part by MYC and Notch signaling, which promotes the dedifferentiation of tumor cells. Our findings suggest that autophagy modulation may influence this plasticity: inhibiting autophagy in NE cells leads to the emergence of non-NE traits, which are often linked to chemoresistance. While our study focused primarily on basal autophagic flux, previous research has shown that chemotherapy often induces autophagy, which can either protect tumor cells or contribute to their death, depending on the context. For example, METTL3-mediated autophagy has been shown to increase chemotherapy resistance (43), while the inhibition of autophagy by statins leads to the accumulation of reactive oxygen species, which sensitizes SCLC cells to treatment (44).

Despite these promising findings, the role of autophagy in SCLC chemoresistance is likely complex and context-dependent. For example, while inhibiting autophagy may decrease NE traits and tumor aggressiveness, it could also impair the autophagic processes that facilitate cell death under stress, potentially limiting the efficacy of chemotherapy. Moreover, autophagy has been shown to play distinct roles at different stages of tumor development. In early stages, it may help tumor cells survive under adverse conditions, whereas in advanced stages, it might promote aggressive behavior and resistance to treatment. These observations suggest that targeting autophagy should be approached with caution, as the timing and context of inhibition could drastically alter therapeutic outcomes.

One limitation of the current study is its reliance on the *RPΔG* genetically engineered mouse model, which primarily recapitulates small cell lung cancer (SCLC) driven by the deletion of RB1 and TP53 genes. While this model captures key genetic and histopathological features of the disease, its relevance to the full complexity of human SCLC remains limited, as the pathogenesis of the disease can also be driven by various other genetic mutations. Therefore, the observed relationship between autophagy and neuroendocrine (NE) characteristics, based solely on this RP-driven SCLC model, should be viewed as an important starting point, providing valuable insights and hypotheses. However, it is essential to consider other SCLC subtypes driven by different mutations in future studies. To better validate and extend our findings, further research utilizing patient-derived xenografts (PDXs) or clinical samples, as well as models incorporating other relevant genetic mutations, will be necessary.

Additionally, the long latency period associated with tumor development in this model limited the number of replicates available for analysis, which could affect the statistical power and robustness of our conclusions. Larger-scale studies with shorter tumor latency, using more diverse models, will help to strengthen and confirm the observed relationships between autophagic flux and tumor progression across different genetic backgrounds.

## 5 Conclusions

The findings derived from the RP conditional knockout mouse model are primarily limited to small cell lung cancer (SCLC) resulting from the deletion of the RB1 and TP53 genes. While this model provides a valuable starting point for exploring the relationship between autophagy and neuroendocrine (NE) characteristics in SCLC, it is important to consider that SCLC may arise from other genetic mutations. Therefore, while the observed relationship between autophagy and NE features offers a promising avenue for further investigation, it may not be universally applicable to all forms of SCLC. More research, including studies on other SCLC models, is needed to confirm and broaden these findings.

## Data Availability

The datasets presented in this study can be found in online repositories. The names of the repository/repositories and accession number(s) can be found in the article/**Supplementary Material**.
