# Supplementary material for: Autophagic flux modulates tumor heterogeneity and lineage plasticity in SCLC
Source: Front Oncol. 2025 Jan 9;14:1509183. doi: 10.3389/fonc.2024.1509183 (PMC11754400; doi:10.3389/fonc.2024.1509183)
Supplement: Supplementary file 1 [file DataSheet1.docx]

**Supplementary Figures**

**
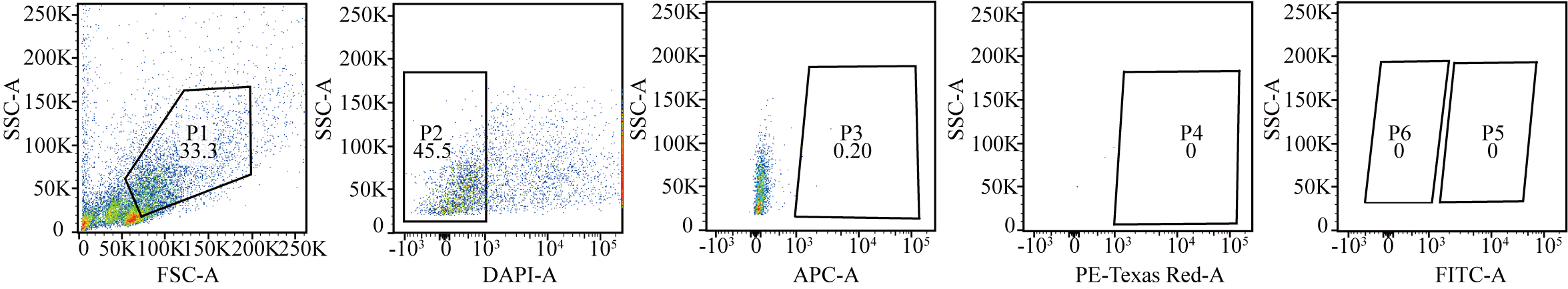
**

**Supplementary Figure 1 |** Flow cytometry gating strategy for lung epithelial cells from wild-type mice (related to Figure 2). Normal lung tissues from wild-type mice were dissociated into single cells for flow cytometry. Debris was excluded, and live cells were gated as DAPI-negative. EpCAM-positive cells were then selected, followed by sorting for RFP-positive cells, and further gating for GFP-positive (GFP+) and GFP-negative (GFP-) subpopulations for RNA sequencing. No RFP-positive cells were found in the wild-type mice, as they lack fluorescent markers.

**
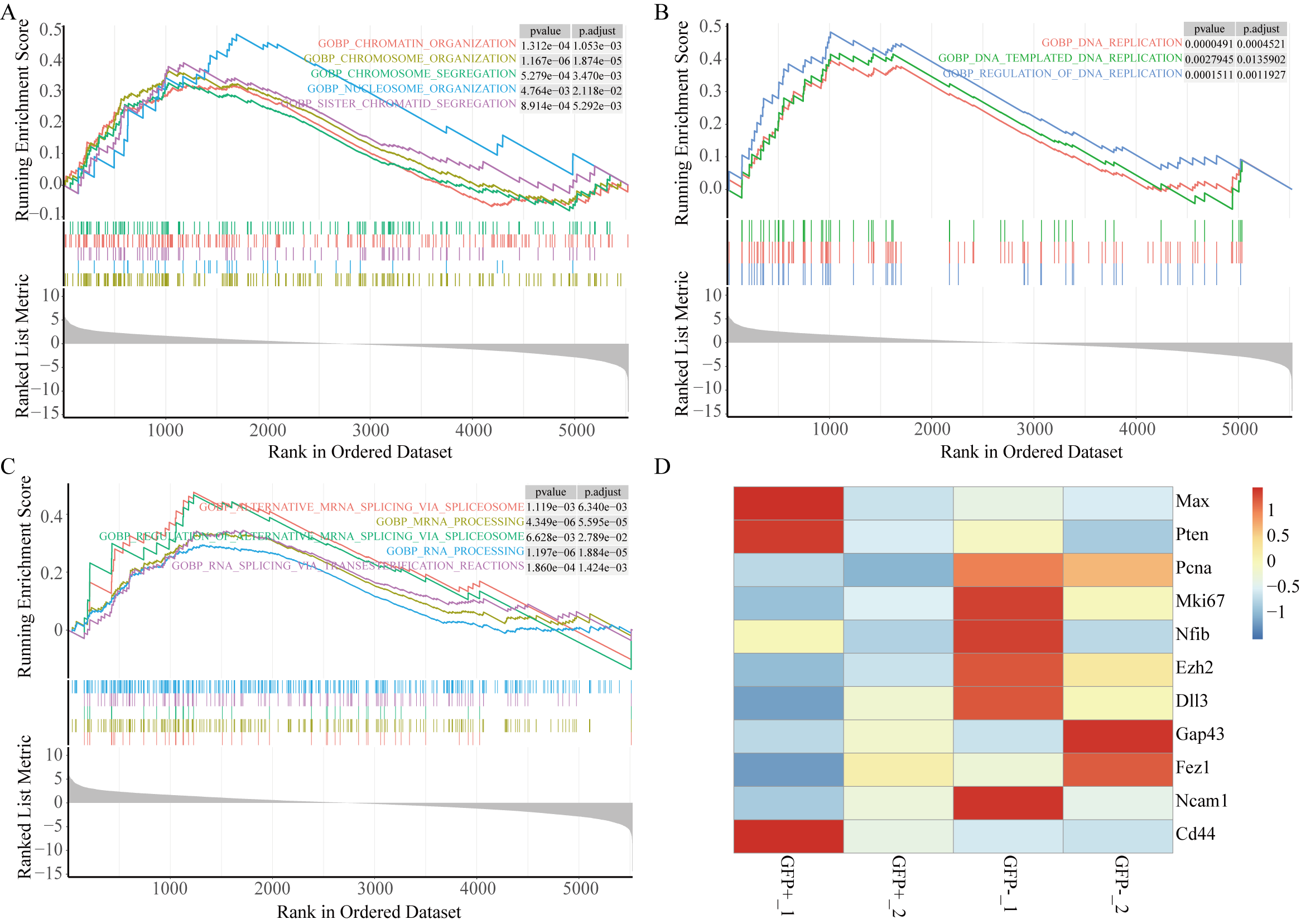
**

**Supplementary Figure 2 |** GSEA of the GFP-negative subpopulation and heatmap of regulatory factors (related to Figure 3). (A-C) GSEA identified enriched pathways in the GFP-negative subpopulation. (A) Chromatin-related pathways. (B) DNA replication pathways. (C) RNA transcription pathways. (D) Heatmap showing validated regulatory factors linked to SCLC progression and metastasis.


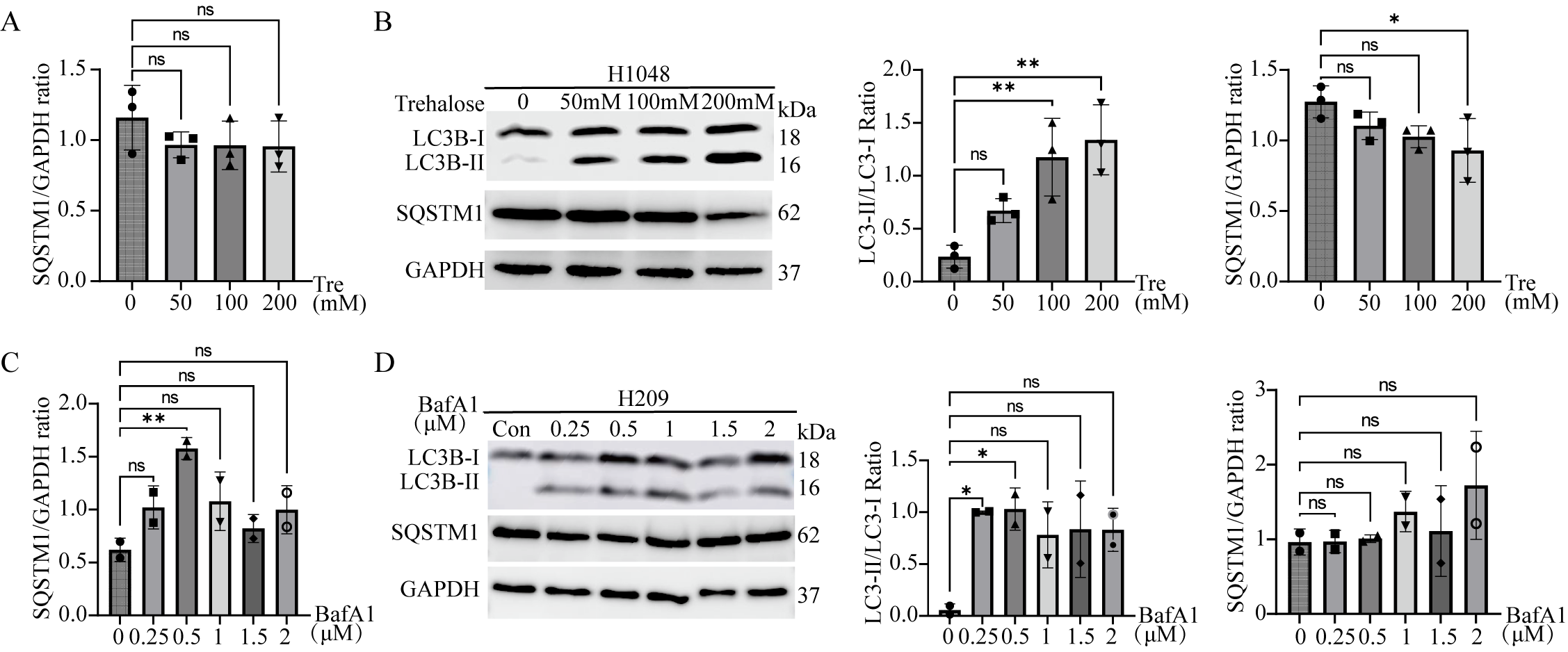


**Supplementary Figure 3 |** Quantification of LC3B and SQSTM1 levels in various SCLC cell lines (related to Figure 5). (A) Western blot analysis of SQSTM1 levels normalized to the loading control GAPDH in H841 cells. The data represent the mean ± SD from three independent experiments. (B) (Left panel) Western blot of LC3B and SQSTM1 in H1048 cells treated with trehalose. (Middle panel) Quantification of the LC3B-II/LC3B-I ratio, used to assess autophagic flux, in H1048 cells. (Right panel) Quantification of SQSTM1 levels normalized to GAPDH in H1048 cells. The data represent the mean ± SD from three independent experiments. (C) Quantification of SQSTM1 levels normalized to GAPDH in H1092 cells. The data represent the mean ± SD from two independent experiments. (D) (Left panel) Western blot of LC3B and SQSTM1 in H209 cells treated with BafA1. (Middle panel) Quantification of the LC3B-II/LC3B-I ratio in H209 cells. (Right panel) Quantification of SQSTM1 levels normalized to GAPDH in H209 cells. The data represent the mean ± SD from two independent experiments.
