# Supplementary material for: Autophagic flux modulates tumor heterogeneity and lineage plasticity in SCLC
Source: Front Oncol. 2025 Jan 9;14:1509183. doi: 10.3389/fonc.2024.1509183 (PMC11754400; doi:10.3389/fonc.2024.1509183)
Supplement: Supplementary file 2 [file DataSheet2.docx]

**Supplementary Tables**

**Supplementary Table 1.** Primers used for the identification of Map1lc3b genome

**Supplementary Table 2**. Primers used for the identification of transgene GFP-LC3-RFP-LC3△G

**Supplementary Table 3.** Primers used for the identification of *Trp53^fl/fl^* and *Rb1^fl/fl^*

**Supplementary Table 4.** Reagents, cell lines and accession numbers

**Supplementary Table 1.** Primers used for the identification of Map1lc3b genome

| sequence 1-F | CTGCGGACTGAGACACACAC |
| --- | --- |
| sequence 1-R | AATATCCCACTGGCTGCGT |
| sequence 2-F | CCATGTTGGTGAGCTCCAC |
| sequence 2-R | CAGTTCAGTCAAGCCCCAG |
| sequence 3-F | CCGTCCTGGACAAGACCAAG |
| sequence 3-R | ACCCACCATGCCTAGCAAACT |
| sequence 4-F | GGATGTCAGACCCTGATCACAG |
| sequence 4-R | AATGTCTCCTGCGAGGCATAA |

**Supplementary Table 2.** Primers used for the identification of transgene GFP-LC3-RFP-LC3△G

| transgene sequence-F | GCCGCCGGGATCACTCTC |
| --- | --- |
| transgene sequence-R | ACCTTCACCTTCCTACGCGTTTAG |

**Supplementary Table 3.** Primers used for the identification of *Trp53^fl/fl^* and *Rb1^fl/fl^*

| Trp53-1 | CGCAATCCTTTATTCTGTTCG |
| --- | --- |
| Trp53-2 | AGCACATAGGAGGCAGAGAC |
| Trp53-3 | TGAGACAGGGTCTTGCTATTG |
| Rb1-F | GGCGTGTGCCATCAATG |
| Rb1-R | CTCAAGAGCTCAGACTCATGG |

**Supplementary Table 4.** Reagents, cell lines and accession numbers

| REAGENT or RESOURCE | SOURCE | IDENTIFIER |
| --- | --- | --- |
| Antibodies |  |  |
| PE anti-DAPI | BD Biosciences | Cat# 564907, RRID: AB_2869624 |
| PE anti-EPCAM | BioLegend | Cat# 118211, RRID: AB_1134104 |
| Mouse anti-SQSTM1 | Abcam | Cat# ab56416, RRID: AB_945626 |
| Rabbit anti-LC3B | Sigma-Aldrich | Cat# L7543, RRID: AB_796155 |
| Mouse anti-GAPDH | Proteintech | Cat# 60004-1-Ig, RRID: AB_2107436 |
| Goat anti-Rabbit IgG (H+L)-HRP Secondary Antibody | Bioworld Technology | Cat# BS13278, RRID: AB_2773728 |
| Goat anti-Mouse IgG (H+L)-HRP Secondary Antibody | Thermo Fisher Scientific | Cat# 31430, RRID: AB_228307 |
| Chemicals and Critical Commercial Assays | | |
| DMEM medium | Gibco | Cat# C11995500BT |
| RPMI 1640 | Gibco | Cat# C11875500BT |
| DMEM/F12 | Meilunbio | Cat# MA0214 |
| Fetal Bovine Serum (FBS) | Gibco | Cat# 10099141C |
| Insulin-Transferrin-Selenium (ITS) | Gibco | Cat# 41400045 |
| Phosphate Buffered Saline (PBS) | Biological Industries | Cat# 02-024-1ACS |
| Optimal Cutting Temperature compound | SAKURA | Cat# 4583 |
| HEPES | Gibco | Cat# 15630-080 |
| Penicillin-Streptomycin-Glutamine (100X) Gibco™ | Gibco | Cat# 15140-122 |
| HBSS | Miltenyi Biotec | Cat#14175-095 |
| DNase I | Sigma-Aldrich | Cat# D4527 |
| TRIzol | Invitrogen | Cat# 98597101 |
| 4% paraformaldehyde | Solarbio | Cat# No. P1110 |
| Trehalose (Tre) | Sigma-Aldrich | Cat# T9531 |
| Bafilomycin A1 (BafA1) | Selleck | Cat# S1413 |
| Ad-CMV-Cre | Vector Biolabs | Cat# 1045 |
| Tumor Dissociation Kit, mouse | Miltenyi Biotec | Cat# 130-100-008 |
| Mouse Direct PCR Kit | Bimake | Cat# B40013 |
| Deposited Data | | |
| RNA-seq of GFP- and GFP+ subpopulation cells | This paper | Accession Number GEO: GSE278235 |
| RNA-seq of primary lung tumors and metastatic liver tumors in *RP* mice | This paper | Accession Number GEO: GSE278236 |
| RNA-seq of H841 and H841-Tre cells | This paper | Accession Number GEO:  GSE284267 |
| RNA-seq of H1092 and H1092-BafA1 cells | This paper | Accession Number GEO: GSE278238 |
| Experimental Cells and Mice Models | | |
| H841 | ATCC | CRL-5845 |
| H1092 | ATCC | CRL-5855 |
| H1048 | ATCC | CRL-5853 |
| H209 | ATCC | HTB-172 |
| GFP-LC3-RFP-LC3ΔG-knockin mice | This paper | N/A |
| *Rb1^fl/fl^; Trp53^fl/fl^* (RP) mice | Gifts from Dr. Hongbin Ji | N/A |
|  | | |
