# Supplementary material for: Autophagic flux modulates tumor heterogeneity and lineage plasticity in SCLC
Source: Front Oncol. 2025 Jan 9;14:1509183. doi: 10.3389/fonc.2024.1509183 (PMC11754400; doi:10.3389/fonc.2024.1509183)
Supplement: Supplementary file 3 [file DataSheet3.docx]

# This code is utilized for the similarity analysis of all bulk RNA-seq datasets.

# Different datasets can be analyzed by simply replacing the GEO data identifiers.

rm(list = ls())

setwd("E:\\GEO data\\GSE183371")

library(readxl)

library(tidyverse)

load("data.Rdata")

exp <- read_csv("GSE183371.csv")

data_new <- merge(data, exp, by.x = 0, by.y = "gene_name")

data_new <- data_new[order(data_new$Row.names), ]

data_new <- data_new[!duplicated(data_new$Row.names), ]

rownames(data_new) <- NULL

data_new <- column_to_rownames(data_new, "Row.names")

data_new <- log2(data_new + 1)

View(data_new)

boxplot(data_new, las = 2, cex.axis = 0.6)

data_new <- scale(data_new)

boxplot(data_new, las = 2, cex.axis = 0.6)

group_list <- data.frame(

sample = colnames(data_new), c(rep("G", 4), rep("1", 6))

)

rownames(group_list) <- group_list$sample

colnames(group_list)[2] <- "dataset"

group <- factor(group_list$dataset)

library(tinyarray)

draw_pca(exp = data_new, group_list = group)

library(sva)

exp_all_combat <- ComBat(data_new, batch = group_list$dataset)

boxplot(exp_all_combat, las = 2, cex.axis = 0.6)

group_list <- data.frame(

sample = colnames(data_new), c(rep("G", 2), rep("Y", 2), rep("1", 6))

)

rownames(group_list) <- group_list$sample

colnames(group_list)[2] <- "dataset"

group <- factor(group_list$dataset)

library(tinyarray)

draw_pca(exp = data_new, group_list = group)

library(sva)

exp_all_combat <- ComBat(data_new, batch = group_list$dataset)

boxplot(exp_all_combat, las = 2, cex.axis = 0.6)

library(corrplot)

library(ggplot2)

library(ggcorrplot)

library(vcd)

library(psych)

library(ggrepel)

dim(exp_all_combat)

data <- as.matrix(exp_all_combat)

data = data.frame(scale(data))

head(data)

data <- cor(data, method = "spearman")

round(data, 2)

data <- cor(data, method = "spearman")

round(data, 2)

pheatmap::pheatmap(data)

ggcorrplot(data, method = "circle")

ggcorrplot(data, method = "circle",

type = "upper",

ggtheme = ggplot2::theme_minimal,

title = "",

show.legend = TRUE,

legend.title = "Corr",

show.diag = TRUE,

colors = c("blue", "white", "red"),

outline.color = "gray",

hc.order = FALSE,

hc.method = "complete",

lab = TRUE,

lab_col = "black",

lab_size = 4,

p.mat = NULL,

sig.level = 0.05,

insig = c("pch", "blank"),

tl.cex = 12,

tl.col = "black",

tl.srt = 45,

digits = 2)

corrplot(data)

corrplot(data, method = "circle",

title = "pearson",

type = "full",

outline = TRUE,

diag = TRUE,

mar = c(0, 0, 0, 0),

bg = "white",

add = FALSE,

is.corr = TRUE,

addgrid.col = "darkgray",

addCoef.col = NULL,

addCoefasPercent = FALSE,

order = "original",

hclust.method = "complete",

addrect = NULL,

rect.col = "black",

rect.lwd = 2,

tl.pos = NULL,

tl.cex = 1,

tl.col = "black",

cl.pos = NULL)

corrplot(data, method = "circle",

title = "",

type = "full",

outline = FALSE,

diag = TRUE,

mar = c(0, 0, 0, 0),

bg = "white",

add = TRUE,

is.corr = TRUE,

addgrid.col = "darkgray",

addCoef.col = "black",

addCoefasPercent = TRUE,

order = "original",

hclust.method = "complete",

addrect = NULL,

rect.col = "black",

rect.lwd = 2,

tl.pos = NULL,

tl.cex = 1,

tl.col = "black",

cl.pos = NULL)

corrplot(data, method = "ellipse", order = "original",

addCoef.col = "black",

type = "full",

title = " Ellipses with black coefficient values",

add = FALSE,

diag = TRUE,

tl.cex = 1,

tl.col = "black",

cl.pos = NULL,

mar = c(1, 1, 1, 1))

corrplot(data, method = "ellipse", order = "original",

addCoef.col = "black",

addCoefasPercent = TRUE,

type = "full",

title = " Ellipses with black percentages",

add = FALSE,

diag = TRUE,

tl.cex = 1,

tl.col = "black",

cl.pos = NULL,

mar = c(1, 1, 1, 1))
